# Cutis Marmorata Telangiectatica Congenita: Case Series and Literature Review

**DOI:** 10.3390/diagnostics15162043

**Published:** 2025-08-14

**Authors:** Mărioara Boia, Daniela-Eugenia Popescu, Ana Maria Cristina Jura, Valerica Belengeanu, Nicoleta Lungu, Aniko Maria Manea, Florina Stoica, Corina Pienar, Eugen Radu Boia

**Affiliations:** 1 Department of Obstetrics and Gynecology, “Victor Babeş” University of Medicine and Pharmacy, Eftimie Murgu Sq. No. 2, 300041 Timişoara, Romania; 2 Medici’s MedLife Hospital Timișoara, Ciprian Porumbescu Street No. 9, 300237 Timișoara, Romania; 3 Department of Medicine, Faculty of Medicine, “Vasile Goldiș” Western University of Arad, 94-96 Revolutiei Blvd., 310025 Arad, Romania; 4 Department of Ophthalmology, Emergency Municipal Clinical Hospital, Gheorghe Dima Street 5, 300254 Timisoara, Romania; 5 Department of Pediatrics, 2nd Pediatrics Clinic, “Victor Babes” University of Medicine and Pharmacy, 300041 Timisoara, Romania; 6 Department of Ear, Nose and Throat, Faculty of Medicine, “Victor Babeș” University of Medicine and Pharmacy Timisoara, 2 Eftimie Murgu Sq., 300041 Timisoara, Romania

**Keywords:** cutis marmorata telangiectatica congenita, vascular malformation, persistent fetal vasculature, neonatal dermatology, congenital anomaly

## Abstract

**Background:** Cutis marmorata telangiectatica congenita (CMTC) is a rare congenital vascular anomaly characterized by a persistent, violaceous, reticulated skin pattern. It may present as a benign isolated lesion or as part of a broader syndrome with systemic anomalies such as limb asymmetry, glaucoma, or neurological impairment. **Methods:** We report a case series of three neonates with CMTC, each representing a distinct clinical pattern: localized, segmental, and generalized. All patients underwent comprehensive clinical assessment, including dermatologic, neurologic, and ophthalmologic evaluations. Additionally, a systematic literature review was conducted using PubMed, Scopus, and Web of Science databases, covering publications from 2012 to 2025. **Results:** Case 1 involved a localized lesion of the calf; Case 2 had segmental involvement of the forearm and leg; Case 3 presented with generalized CMTC covering over 85% of the body surface, accompanied by dysmorphism and bilateral persistent fetal vasculature (PFV). Literature findings highlighted significant clinical variability and a stronger association of generalized forms with systemic abnormalities. **Conclusions:** CMTC exhibits a broad clinical spectrum. While localized cases often resolve spontaneously, generalized forms may require multidisciplinary evaluation. Early recognition and systemic screening are crucial for optimal management.

## 1. Introduction

Cutis marmorata telangiectatica congenita (CMTC) is a rare congenital vascular anomaly involving the capillaries and venules, characterized by a persistent bluish-purple, reticulated skin pattern that resembles physiological cutis marmorata but does not resolve with warming. The condition was first described by Dutch pediatrician Cato van Lohuizen in 1922 [1] and has since remained a diagnostic challenge due to its phenotypic variability and occasional overlap with other syndromic vascular malformations.

CMTC typically manifests at birth or shortly thereafter and may present as localized, segmental, or generalized cutaneous lesions. While many cases are benign and limited to the skin, others may be associated with systemic anomalies, including limb asymmetry, glaucoma, neurological deficits, or skeletal abnormalities [2,3]. The severity of these extracutaneous features often correlates with the extent of skin involvement.

Although the precise etiology of CMTC remains uncertain, several theories have been proposed, including somatic mosaicism, autosomal dominant inheritance with reduced penetrance, and sporadic mutations in genes such as GNA11 and ARL6IP6 [4,5]. These insights have been supported by histological studies and limited genetic investigations, although no definitive pathognomonic genetic marker has been universally confirmed.

The diagnosis of CMTC is established using the major and minor criteria outlined by Kienast and Hoeger. The three primary criteria are as follows: (1) congenital reticular erythema; (2) absence of venectasia in the affected dermis; and (3) persistent erythema that does not resolve with localized warming. Supporting minor criteria include reduction in erythema within the first two years of life, the presence of telangiectasia or port-wine stain, ulceration, and atrophy in the affected region. A diagnosis is considered probable when all three primary criteria and at least two secondary criteria are met. These criteria were applied to each case in the present series [6]. These criteria emphasize the congenital appearance of reticulated erythema, the absence of venectasia in the first year of life, and resistance to warming. Cutis marmorata telangiectatica congenita (CMTC) is an extremely rare vascular anomaly, with fewer than 500 cases reported in the literature worldwide [7].

This report describes three neonatal cases of CMTC, each representing different levels of severity and distribution, from a small localized lesion to widespread involvement with significant ocular and systemic findings. In addition to these case reports, we conduct a comprehensive review of the literature to underscore the clinical variability of CMTC and the importance of thorough systemic evaluation in affected neonates.

## 2. Detailed Case Description

### 2.1. Case Series

This study presents a descriptive case series of three neonates diagnosed with cutis marmorata telangiectatica congenita (CMTC) between January 2022 and December 2024 at a tertiary-care neonatal unit. All cases were diagnosed clinically based on persistent reticulated vascular patterns unresponsive to warming and were confirmed to meet major and minor criteria outlined by Kienast and Hoeger [2]. Physical examination focused on the distribution, depth, and appearance of skin lesions. Systemic evaluations included neurological assessments, cranial ultrasound, echocardiography, and ophthalmological examination using indirect fundoscopy and RetCam imaging where indicated.

Informed written consent was obtained from the parents or legal guardians of each neonate. Ethics approval for this study was obtained from the Institutional Review Board of the Clinical Emergency Hospital for Children “Louis Țurcanu” Timișoara (Approval Code: 9429/10.06.2025), and the research was conducted in accordance with the Declaration of Helsinki.

Clinical photographs were taken to document lesion appearance and progression. Data on birth history, maternal health, family history, and perinatal risk factors were also collected. Follow-up was maintained post-discharge to monitor lesion evolution and potential complications.

### 2.2. Literature Review

In addition to the case series, a systematic literature review was performed to contextualize our findings and evaluate the broader clinical landscape of CMTC, particularly focusing on neonatal presentations and associated systemic anomalies.

#### Search Strategy

**Figure 1:**
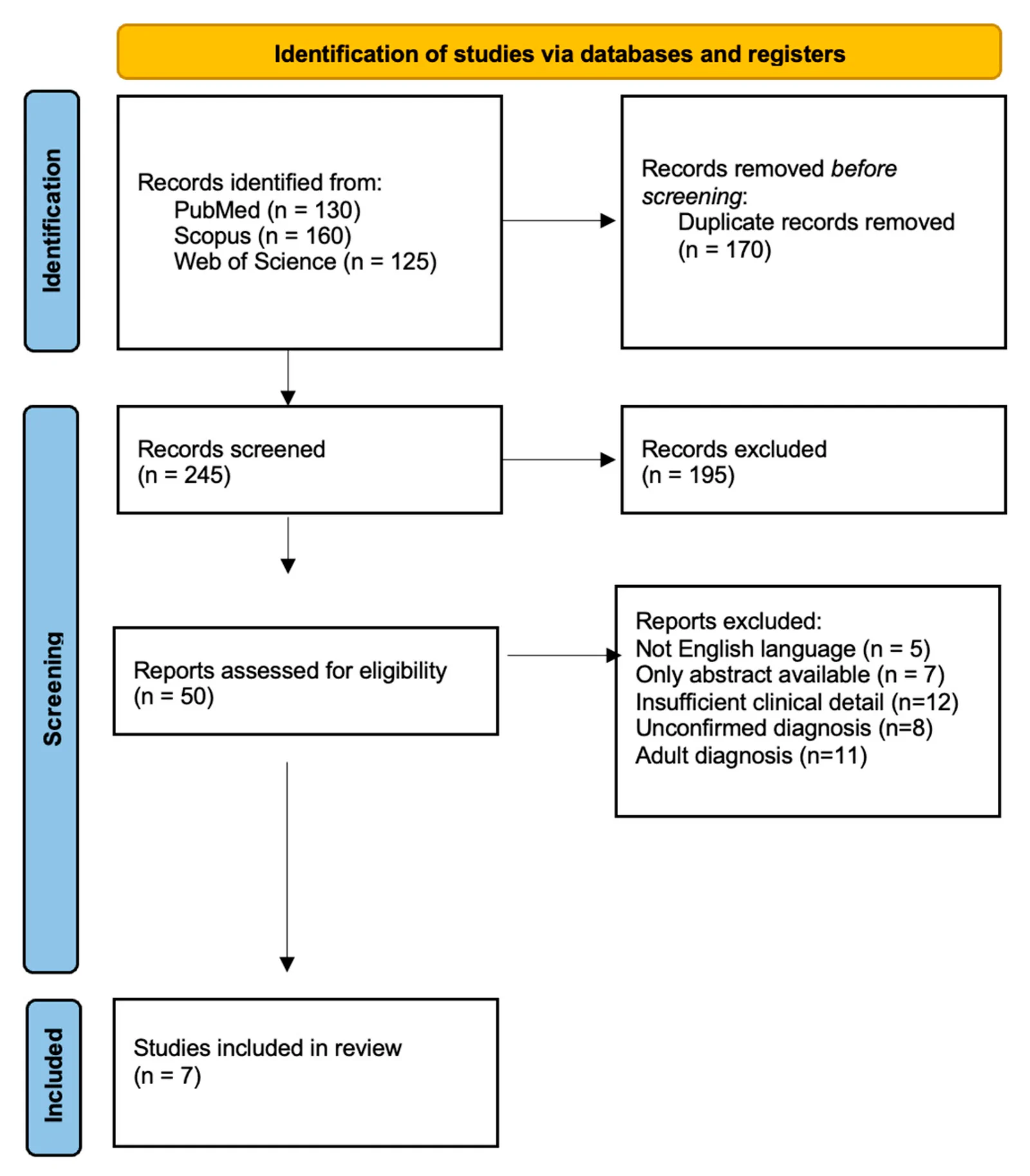
Flowchart for study selection according to PRISMA 2020 guidelines.

We conducted a systematic search of PubMed, Scopus, and Web of Science for case reports and series on neonatal CMTC published from January 2012 to April 2025. Our search strategy included the terms “Cutis marmorata telangiectatica congenita” OR “CMTC”, “Congenital vascular anomaly”, “Neonatal dermatology”, “Persistent fetal vasculature” “Infantile hemangioma”, AND “Systemic anomalies in CMTC”, in English. The review included studies published from January 2010 to April 2025, in English, focusing on neonates or pediatric patients diagnosed with CMTC. We examined reference lists of relevant articles and reviewed conference abstracts for additional cases, following PRISMA 2020 guidelines [8]. Reports eligible for inclusion had to present primary clinical data on CMTC diagnosed during the neonatal period; those lacking clinical specifics, animal studies, or reviews were excluded. Two reviewers (AMCJ and DEP) independently evaluated titles, abstracts, and full texts, resolving discrepancies through consensus. The search and selection process is summarized in Figure 1.

Inclusion criteria: Articles describing CMTC in neonates or children, with sufficient clinical detail, photographs or imaging, and systemic assessments. Exclusion criteria: non-English publications, articles lacking patient-level data, conference abstracts without peer review, and duplicate studies.

Data extracted from each study included patient age at diagnosis, lesion distribution (localized, segmental, or generalized), presence of systemic anomalies (neurological, ocular, skeletal, endocrine), diagnostic criteria used, genetic testing, and outcome or follow-up data. Findings were synthesized narratively and, where applicable, are presented in tabular format to compare patterns and outcomes across cases.

This approach allowed integration of both original clinical observations and published evidence to better understand the clinical heterogeneity, diagnostic criteria, and prognostic implications of CMTC in neonates.

### 2.3. Case 1: Localized CMTC

**Figure 2:**
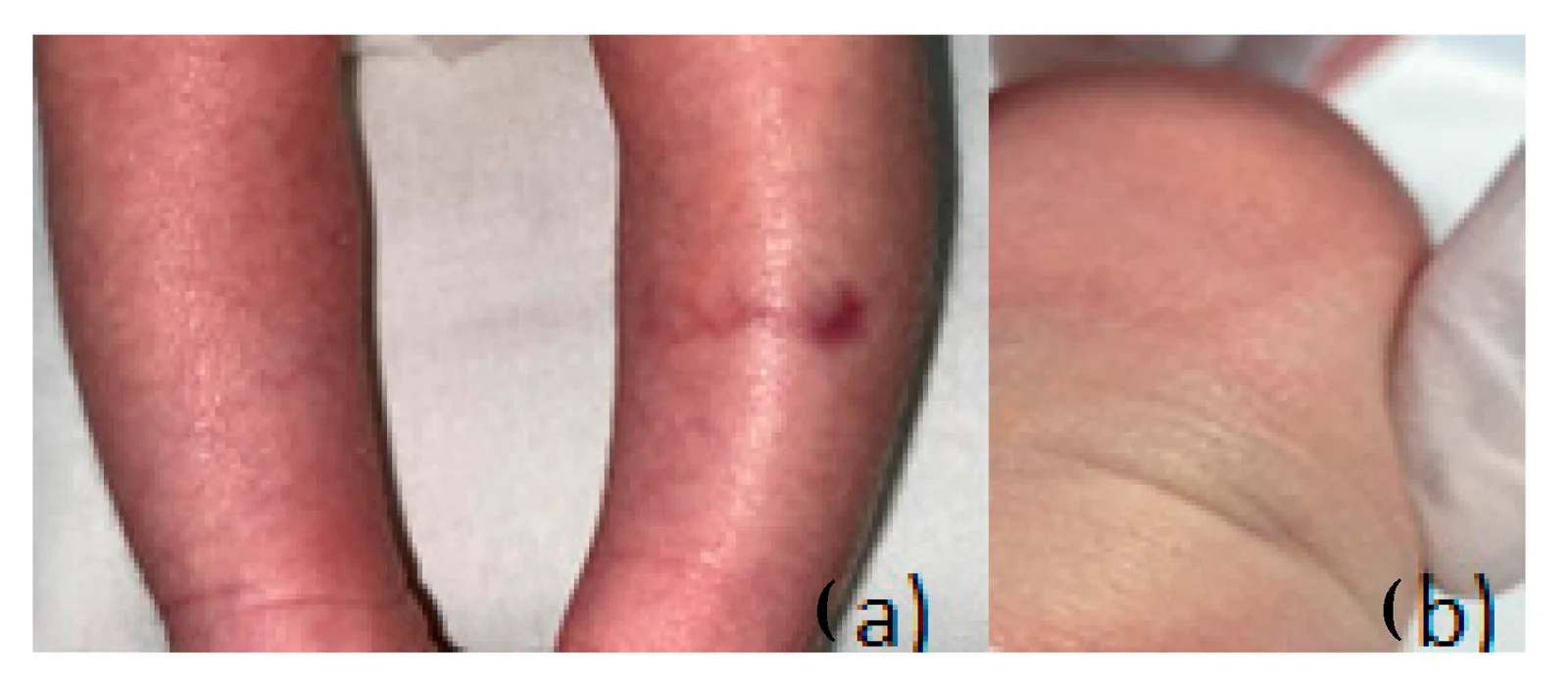
(**a**) Reticulated violaceous lesion on the lateral aspect of the left calf. (**b**) Subcutaneous atrophy with reduced calf circumference.

**Figure 3:**
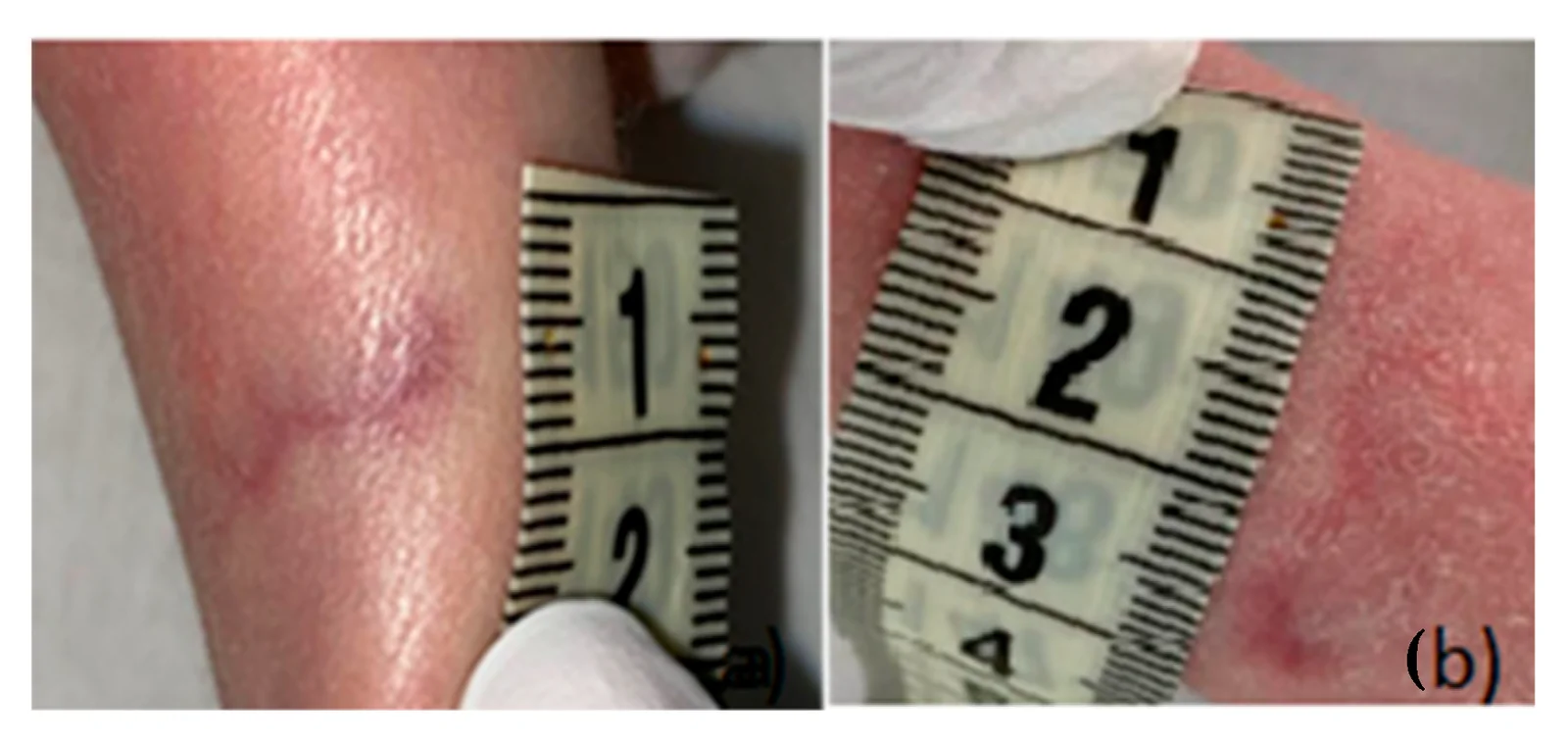
(**a**) Lesion with 0.8 cm in width; (**b**) lesion with 2.5 cm in length.

**Figure 4:**
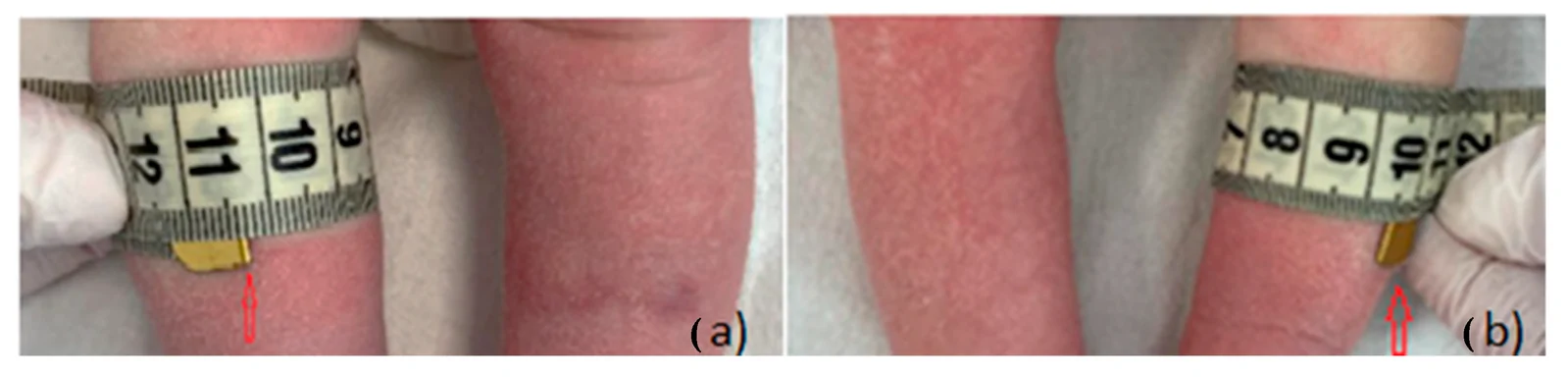
(**a**) The diameter of the right calf; (**b**) the diameter of the left calf.

A female neonate, born at 39 weeks via elective cesarean section after in vitro fertilization, presented with intrauterine growth restriction (birth weight: 2200 g). Apgar scores were 8 and 9 at 1 and 5 min, respectively. On clinical examination, a sharply demarcated violaceous linear lesion measuring approximately 0.8 × 2.5 cm was observed on the lateral aspect of the left calf. The lesion (Figure 2, Figure 3 and Figure 4) was reticulated, non-blanching, and did not resolve with warming. Subcutaneous atrophy was evident, and the calf circumference was reduced on the affected side (left 9.5 cm vs. right 10.2 cm).

Ophthalmologic examination ruled out glaucoma or retinal pathology. Echocardiography showed a patent foramen ovale. Thyroid function tests were normal. The lesion remained stable during early follow-up.

### 2.4. Case 2: Segmental CMTC

**Figure 5:**
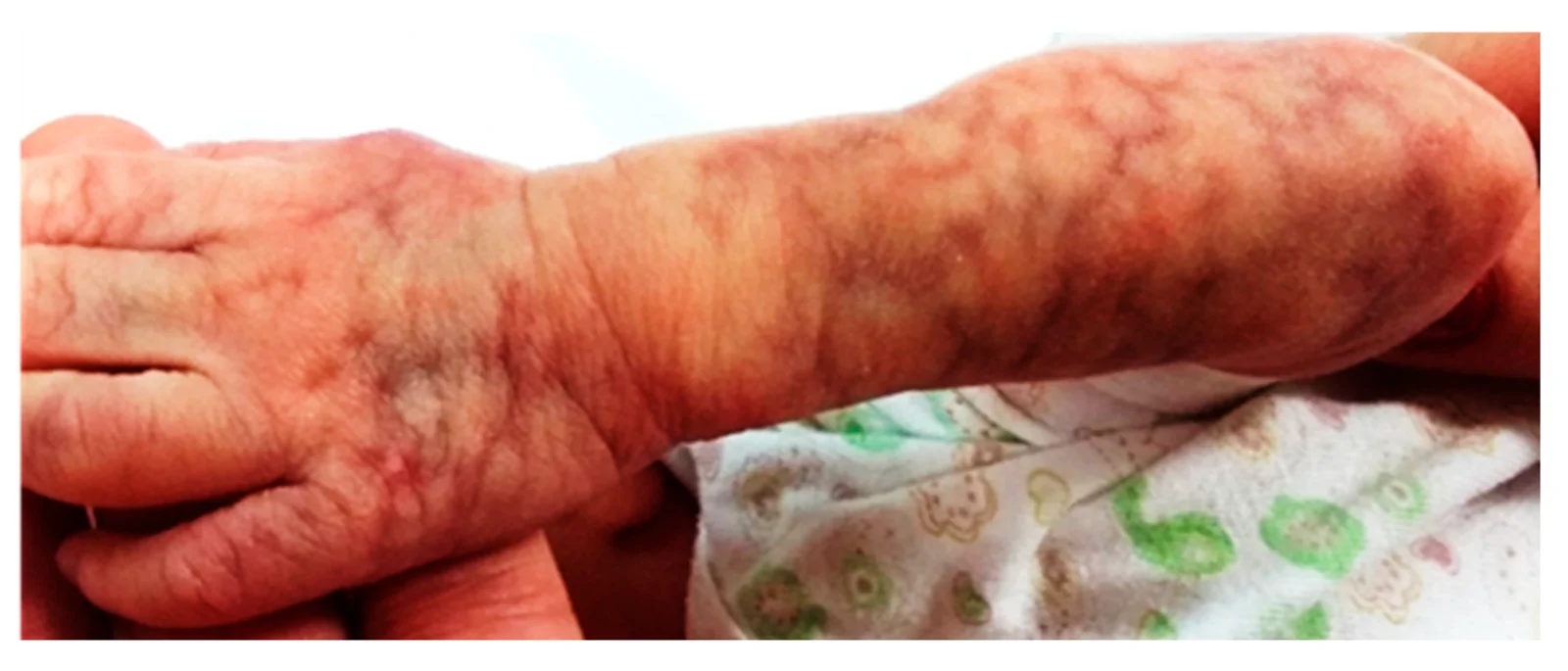
Segmental marbled pattern on the left forearm.

**Figure 6:**
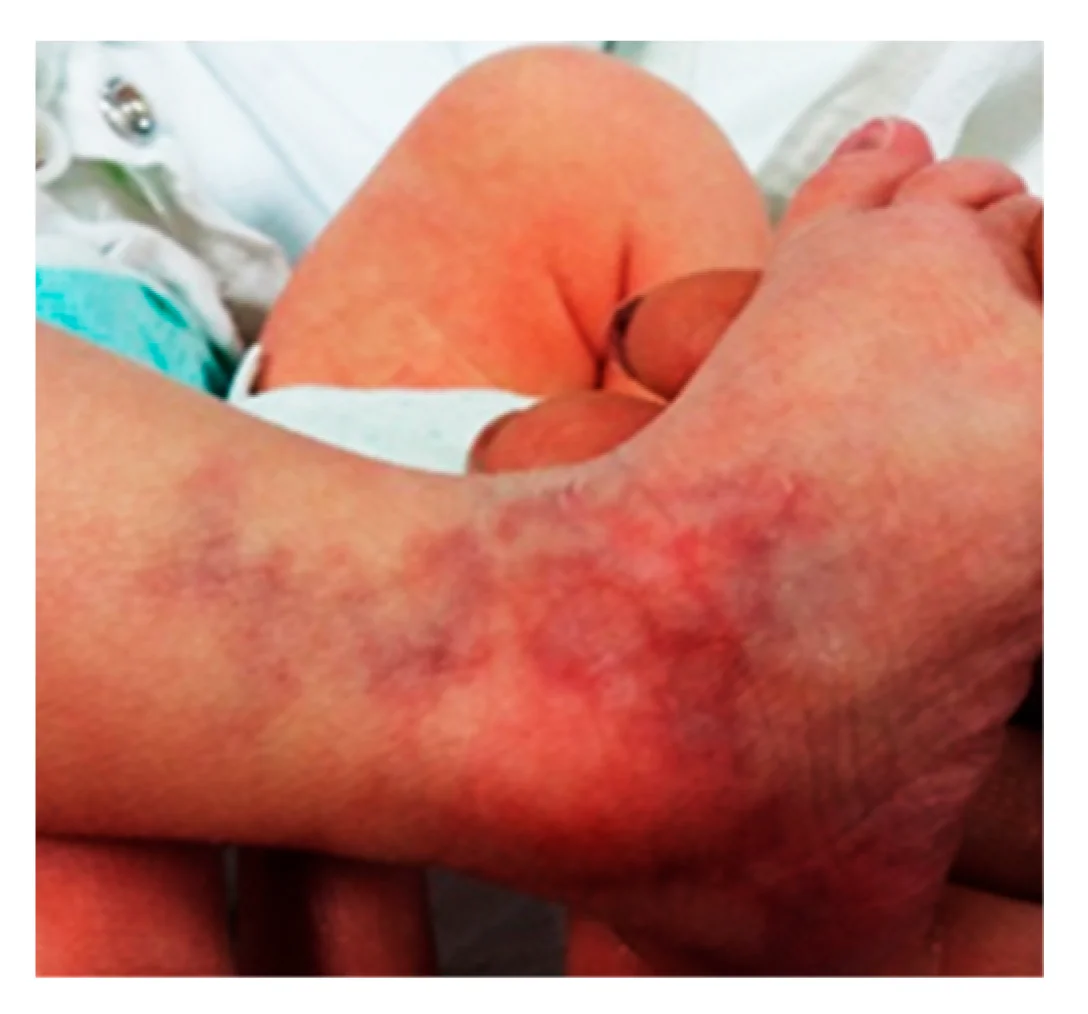
Large violaceous plaque with telangiectasia and mild atrophy on the right leg.

A full-term male neonate born to non-consanguineous parents via cesarean section presented with persistent, segmental vascular lesions on the left forearm and the lateral aspect of the right lower leg. Apgar scores were 8 and 9, respectively. The vascular lesions had a marbled appearance and measured approximately 6 × 11 cm. They were non-blanchable and unresponsive to warming. Mild telangiectasia and focal skin atrophy were noted (Figure 5 and Figure 6).

The patient had a brief episode of cyanosis on day 1, requiring supplemental oxygen for 12 h. Cranial ultrasound revealed mild periventricular hypoxic-ischemic changes. No other systemic abnormalities were identified. The skin lesions remained stable without ulceration during outpatient follow-up.

### 2.5. Case 3: Generalized CMTC with Bilateral Persistent Fetal Vasculature

**Figure 7:**
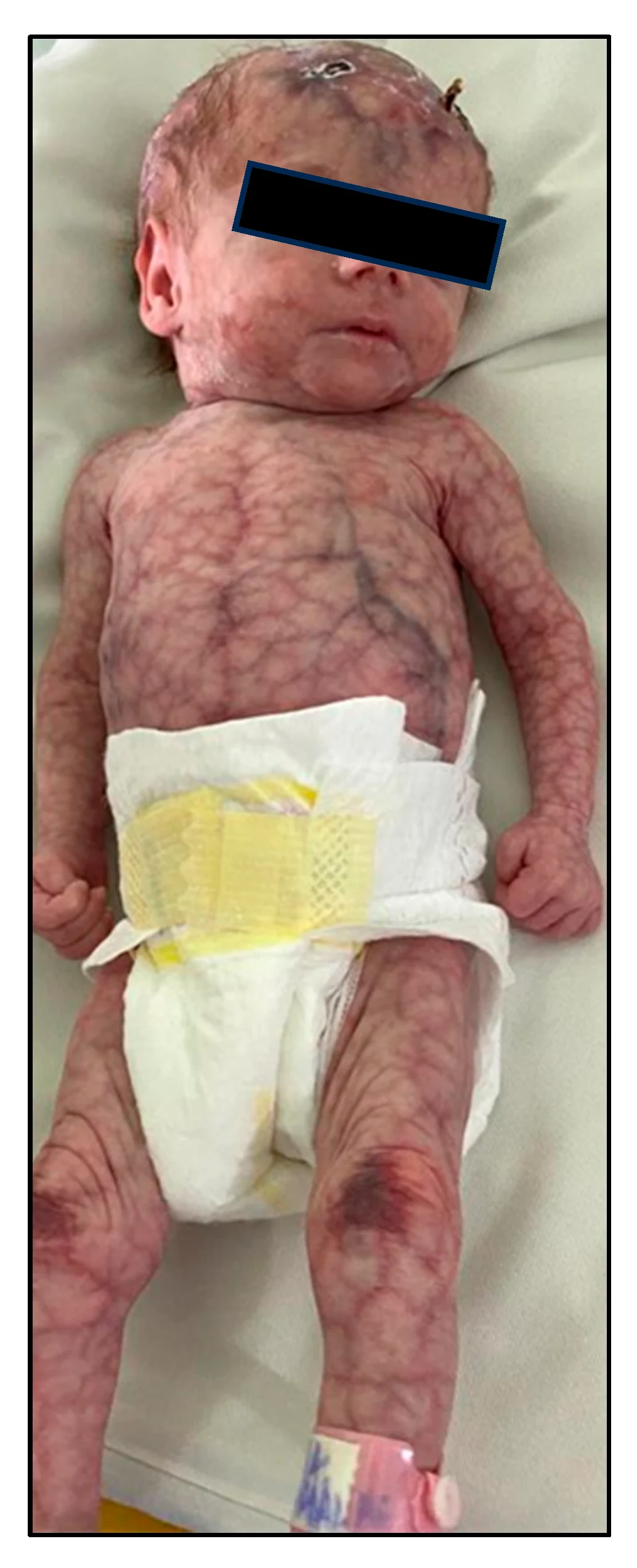
Extensive reticulated purplish pattern over trunk and limbs.

**Figure 8:**
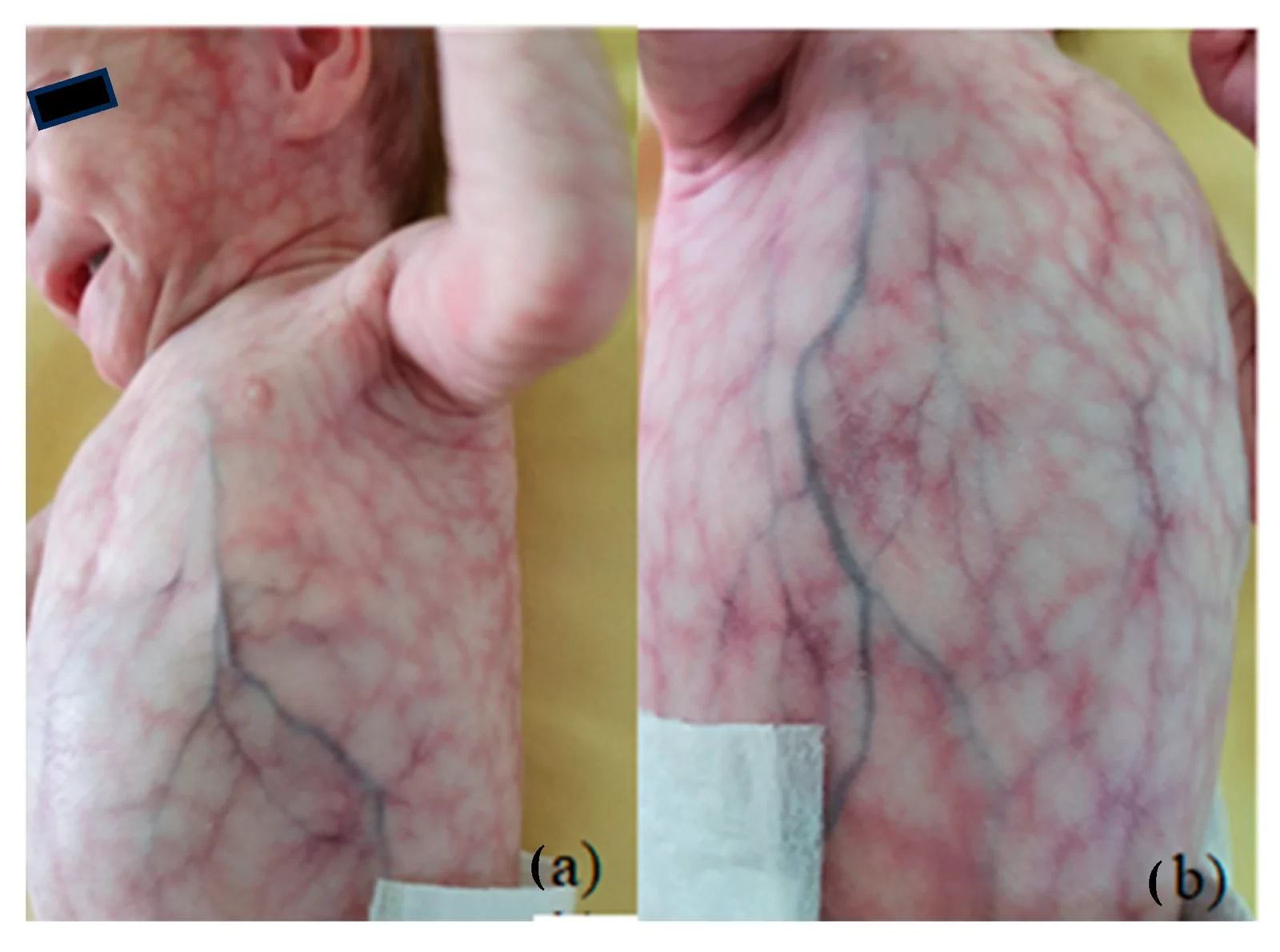
Dysmorphic facial features with visible phlebectasia. (**a**) Phlebectasia in the left part of thorax; (**b**) Phlebectasia in the right part of thorax.

**Figure 9:**
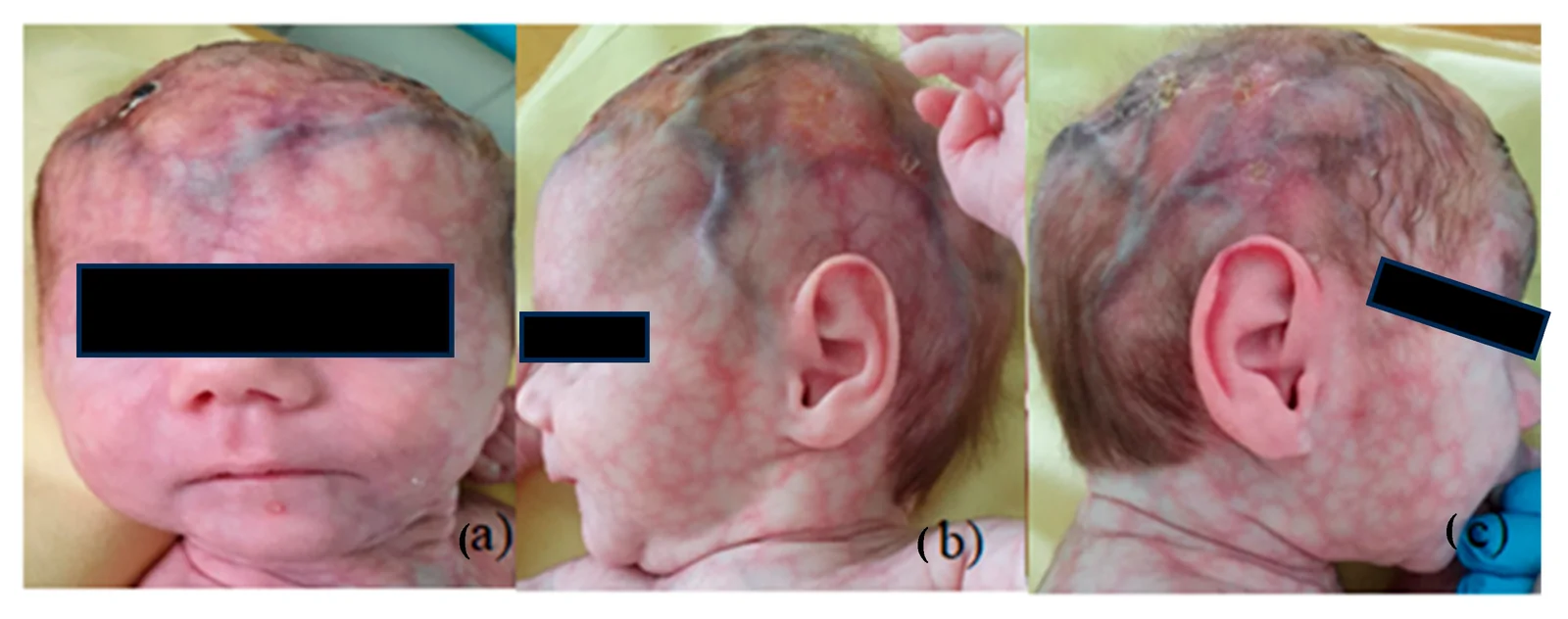
(**a**) Phlebectasia in the front and facial dysmorphism; (**b**) phlebectasia in the fronto-parietal region and microretrognathia; (**c**) phlebectasia in the occipital region, short neck.

A preterm female neonate (gestational age 35 weeks, birth weight 1450 g) was admitted to the NICU for respiratory distress and low birth weight. She had a striking generalized violaceous reticulated skin pattern covering approximately 85% of her body surface area, including the scalp, face, trunk, and limbs. Lesions were deep purple, fixed, and associated with phlebectasia and scaling. Ulceration was noted on the thoracic region and knees (Figure 7, Figure 8 and Figure 9).

The patient also exhibited dysmorphic facial features including microphthalmia, hypertelorism, low-set ears, and microretrognatia. Neurological evaluation revealed generalized hypotonia. Cranial ultrasound showed grade II intraventricular hemorrhage and signs of hypoxic-ischemic encephalopathy.

**Figure 10:**
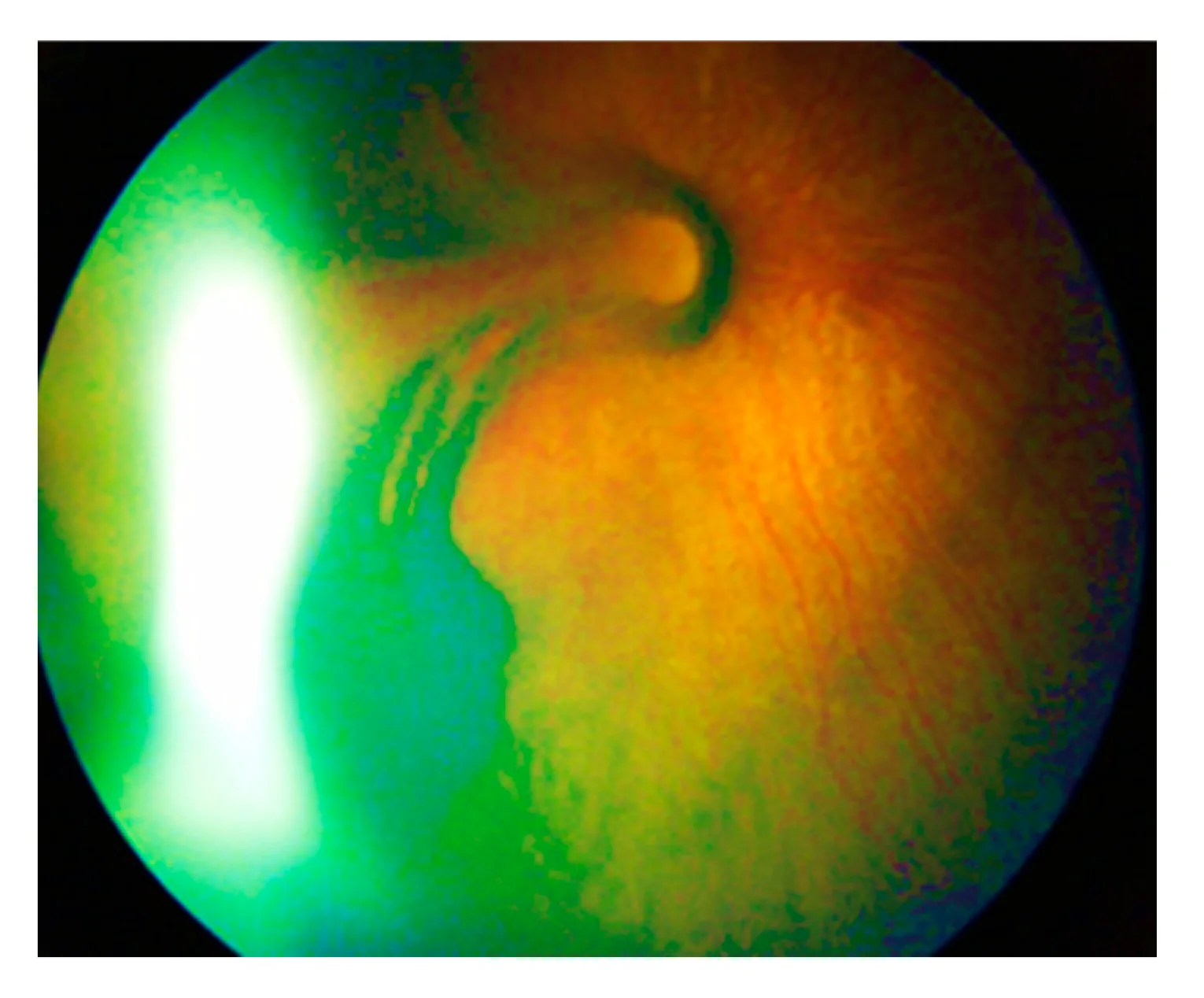
RetCam image showing PFV with fibrovascular stalks and retinal distortion.

Ophthalmologic assessment via RetCam imaging identified bilateral persistent fetal vasculature (PFV), with retinal folds, microphthalmia, and fibrovascular stalks extending from the optic disc to the posterior lens capsule, more visible in the left eye (Figure 10).

The infant’s clinical condition deteriorated due to neonatal sepsis, and she succumbed at 8 days of life. Genetic testing could not be performed prior to her demise.

Detailed clinical information for all three cases, including perinatal history, lesion characteristics, associated anomalies, neurological and ophthalmologic findings, and outcomes, is summarized in Table 1. In Cases 1 and 2, the vascular biopsy required for genetic diagnosis was not performed due to parental refusal during the neonatal period. Testing was not performed for Case 3 due to the patient’s rapid clinical deterioration and death.

The literature review table (Table 2) consolidates representative case reports and case series of CMTC published between 2012 and 2025. It highlights important trends in clinical presentation, gestational age, lesion distribution, associated anomalies, and outcomes.

The cases reflect that CMTC can occur in both term and preterm neonates, though more generalized forms appear slightly more common among preterm infants. For instance, both refs. [9,13] reported generalized presentations in preterm neonates, which were associated with systemic complications such as dysmorphism, hypoxic-ischemic encephalopathy (HIE), or ocular anomalies like glaucoma. These findings suggest that earlier gestational age may correlate with a more widespread or syndromic disease expression, possibly due to immature vascular remodeling.

Lesion distribution ranged from localized to generalized. Localized forms, such as that reported by [6], typically involved a single limb and were associated with milder complications, such as limb hypotrophy. In contrast, generalized or multi-segmental lesions were more often seen in the more severe cases [11,12] some of which required long-term monitoring due to persistent skin changes and potential underlying anomalies. The variability in distribution reflects the theory that CMTC may represent a spectrum disorder influenced by the degree and timing of vascular dysregulation during embryogenesis.

Systemic involvement was found in more than half of the reviewed cases, consistent with the existing literature. Common anomalies included limb asymmetry or hypotrophy [6], ophthalmologic abnormalities such as unilateral glaucoma ([9]), and neurological findings including hypoxic-ischemic encephalopathy (HIE) and dysmorphism [13].

These associations reinforce the recommendation for a multidisciplinary evaluation in all cases of CMTC, especially when the lesions are extensive, facial, or accompanied by dysmorphic features. Notably, some cases—such as those by [11,12]—had no detectable anomalies, underscoring the existence of a benign subset.

Clinical outcomes varied. Localized lesions, as in the Resende case, showed improvement over time with some residual asymmetry. Generalized lesions had less predictable outcomes, with some showing stabilization or partial regression. Long-term prognostic data remains sparse due to limited follow-up durations in many reports. However, the spontaneous fading of cutaneous lesions by age 2, reported in several studies, supports a conservative management approach in uncomplicated cases.

This aggregation of cases illustrates the need for early identification and systemic screening in neonates with CMTC. The table underscores how lesion distribution and gestational age may help predict associated anomalies and guide monitoring. Incorporating imaging (e.g., cranial ultrasound, echocardiography, ophthalmologic assessment) is particularly important for generalized or facially involved cases. The relatively favorable outcomes in localized cases support a reassuring prognosis, though vigilance remains essential due to the possibility of late-onset manifestations.

## 3. Discussion

Cutis marmorata telangiectatica congenita (CMTC) represents a rare but clinically significant vascular anomaly that can range from isolated cutaneous involvement to complex syndromic presentations. Our three cases exemplify this clinical spectrum—from a solitary, linear lesion confined to the calf, to a generalized and multisystemic form associated with dysmorphism and persistent fetal vasculature (PFV). These variations reaffirm CMTC as a heterogeneous condition with diverse implications for diagnosis, prognosis, and management.

The generalized presentation of Case 3, characterized by extensive reticulated erythema, limb asymmetry, dysmorphic facial features, bilateral persistent fetal vasculature (PFV), and intracranial hemorrhage, exemplifies one of the most severe neonatal CMTC phenotypes documented. Ref. [13] documented a neonate with generalized CMTC, hydrocephalus, limb asymmetry, and PFV; ref. [9] reported a preterm infant with generalized lesions, midline facial anomalies, and PFV. Similarly to our case, both reports observed significant ocular anomalies and neurological deficits, indicating that generalized CMTC may correlate with an increased risk of systemic malformations and adverse neurodevelopmental outcomes. We noted that PFV led to advancing ocular complications necessitating enucleation, highlighting the importance of prompt ophthalmologic monitoring.

Prognosis and treatment: CMTC lesions generally diminish in pigmentation during the initial two years of life but rarely resolve entirely; thus, conservative management is the standard approach [14]. Parents should be advised to prevent cold exposure and trauma to the affected regions, as cyanosis may worsen with vasoconstriction. Ulcerations require immediate treatment, and limb asymmetry should be monitored; surgical interventions are infrequently necessary. Laser therapy typically results in minimal improvement. The long-term prognosis is favorable for localized cases, although limb length discrepancy may persist. In contrast, generalized CMTC associated with systemic anomalies, as demonstrated in Case 3 and the studies by [9,13], may present a cautious prognosis, underscoring the necessity for multidisciplinary monitoring [9].

The diagnosis of CMTC remains primarily clinical. Hallmark features include a marbled, violaceous skin pattern that does not resolve with warming, and is often present from birth. Kienast and Hoeger’s diagnostic criteria [2] are widely accepted and were met in each of our cases. While imaging and histopathology may be helpful in select instances, especially when differentiating from syndromic vascular malformations, most cases do not require invasive diagnostics.

The generalized form, as seen in our third case, often raises suspicion of a syndromic or systemic condition. Generalized CMTC has been associated with significant comorbidities such as ocular defects, central nervous system abnormalities, and dysmorphism [3,4]. Our patient’s bilateral PFV is a rare association, previously reported only in isolated cases such as those by [15], and may indicate an expanded phenotypic spectrum.

Up to 50% of CMTC patients present with associated anomalies, particularly in cases with widespread cutaneous involvement. These may include neurological impairments (e.g., hypotonia, cerebral hemorrhage); ophthalmologic conditions, such as glaucoma or PFV; limb asymmetry, atrophy, or hypertrophy; and ulcerations, which may lead to infection. CMTC should be distinguished from conditions like physiologic cutis marmorata, which resolves with warming; Klippel–Trenaunay syndrome (associated with limb hypertrophy and venous malformations); Adams–Oliver syndrome, especially in cases with limb defects and aplasia cutis; and Phakomatosis pigmentovascularis, involving concurrent vascular and pigmentary nevi [16,17,18].

Although CMTC is easily identifiable by its distinctive reticulated violaceous pattern and congenital onset, it is crucial to differentiate it from physiologic cutis marmorata and other vascular anomalies [19]. Unlike physiologic cutis marmorata, CMTC remains visible despite warming and is often linked to localized atrophy or ulceration. In contrast to Klippel–Trenaunay Syndrome (KTS), marked by venous malformations, limb hypertrophy, and port-wine stains, CMTC does not show notable venous dilation or deep tissue overgrowth in the neonatal phase [14]. Furthermore, CMTC lesions are static, while infantile hemangiomas have a distinct proliferative phase followed by involution [20]. Capillary malformations (port-wine stains), which can be mistaken for CMTC, are flat, non-reticulated, and often exhibit segmental distribution, sometimes associated with syndromes like Sturge–Weber. Key differentiators include the lesion’s morphology and evolution, accompanying systemic findings and temperature responsiveness. A thorough clinical assessment is essential, and, in uncertain cases, interdisciplinary collaboration may help clarify the diagnosis and guide treatment.

Recent insights from genetic studies have pointed to somatic mosaicism and mutations in GNA11 and ARL6IP6 as possible contributors [7,21]. However, genetic testing is still not routine in clinical practice and is typically reserved for complex or recurrent cases.

Severe neonatal dermatological presentations may warrant a broader genetic workup, as illustrated by Matyas et al., who reported a rare co-occurrence of ITGB4 and KRT10 mutations in a neonate with epidermolysis bullosa, pyloric atresia, and aplasia cutis congenita, a combination highlighting the complex interplay of structural skin proteins and congenital anomalies [22].

Though distinct in clinical presentation, CMTC shares overlapping features with other vascular anomalies, particularly infantile hemangiomas. In a 2023 cross-sectional study, Sandru et al. [23] emphasized the diagnostic variability of infantile hemangiomas and their systemic associations, such as airway obstruction and PHACES syndrome. While CMTC lesions are static and present at birth, infantile hemangiomas tend to proliferate after birth and later involute. Nonetheless, both conditions highlight the importance of early dermatological evaluation and awareness of associated risks.

In evaluating neonates with CMTC, particularly those with dysmorphic features or systemic anomalies, screening for endocrine abnormalities is also prudent. Nastase et al. [24] reported two cases of congenital hypothyroidism due to thyroid agenesis, one of which presented with cutaneous vascular findings. While none of our cases exhibited thyroid dysfunction, their report supports the inclusion of thyroid screening in neonates with generalized or syndromic CMTC to avoid delayed diagnosis and neurodevelopmental compromise.

Management of CMTC is largely conservative, with most cases requiring only observation and routine follow-up. Skin care should be gentle, and secondary infections must be prevented, particularly in ulcerated lesions. Generalized cases or those with systemic involvement require coordinated care from dermatology, neurology, ophthalmology, and genetics teams.

Although localized CMTC often resolves within the first 2 years of life, systemic features may persist and require long-term monitoring. The presence of PFV in our third case, along with dysmorphism and prematurity, suggests a potentially novel syndromic variant of CMTC, reinforcing the need for broader phenotypic recognition and potentially expanded diagnostic criteria in the future.

## 4. Conclusions

Cutis marmorata telangiectatica congenita (CMTC) is a rare vascular anomaly with variable presentations, from localized skin lesions to generalized forms with systemic involvement. Our cases highlight the importance of clinical diagnosis and the need for thorough evaluation in severe presentations. While most cases resolve spontaneously, generalized or syndromic forms may require multidisciplinary follow-up. Increased awareness and early identification are key to optimizing outcomes in affected neonates.

## Figures and Tables

**Table 1:** Summary of cases.

Parameter	Case 1	Case 2	Case 3
Sex	Female	Male	Female
Gestational age	39 weeks	40 weeks	35 weeks
Birthweight	2200 g	2850 g	1450 g
Maternal factors	IVF pregnancy, elective C-section	None reported	Premature rupture of membranes
Family history	Negative	Negative	Negative
Lesion distribution	Lateral left calf	Left forearm, right lower leg	Generalized (scalp, face, trunk, limbs)
Lesion characteristics	0.8 × 2.5 cm, reticulated, non-blanching, subcutaneous atrophy, reduced calf circumference	6 × 11 cm marbled lesions, mild telangiectasia, focal atrophy	Fixed reticulated pattern over 85% BSA, phlebectasia, scaling, ulceration
Associated anomalies	None	Transient cyanosis, patent foramen ovale	Dysmorphism, ulcerations, bilateral PFV, HIE, IVH
Neurological findings	Normal	Mild periventricular HIE on ultrasound	Hypotonia, HIE, IVH grade II
Ophthalmologic findings	Negative vascular anomaly panel	Negative vascular anomaly panel	Not performed
Outcome	Lesion stable on follow-up	Lesion stable, no ulceration	Died on day 8 due to sepsis

**Table 2:** Summary of published neonatal CMTC cases (2012–2025).

Reference	Gestational Age	Lesion Distribution	Associated Anomalies	Outcome
Resende et al. [6]	Full-term	Left upper limb	Limb hypotrophy	Lesions improved with age; limb asymmetry persisted
De Maio et al. [9]	Preterm	Generalized	Unilateral congenital glaucoma	Favorable
Leung et al. [10]	Not specified	Right lower limb	Ipsilateral hemiatrophy	Not specified
Portal Buenaga et al. [11]	Term	Lower extremities, upper extremity, hypochondrium, lumbosacral region	None	Partial resolution at 4 months
Manna S. et al. [12]	Full-term	Trunk, limbs, face	None	Not specified
Matic et al. [13]	Preterm	Generalized	Dysmorphisms, micrognathia, low-set ears, visual delay	Not specified
Amaral et al. [5]	Term	Right-sided segmental	ASD, right heart dominance, limb asymmetry	Not specified

## Data Availability

The original contributions presented in this study are included in the article. Further inquiries can be directed to the corresponding authors.

## Abbreviations

The following abbreviations are used in this manuscript:

CMTC	Cutis marmorata telangiectatica congenita
PFV	Persistent fetal vasculature
NICU	Neonatal intensive care unit
HIE	Hypoxic-ischemic encephalopathy
IVH	Intraventricular hemorrhage
IVF	In vitro fertilization
BSA	Body surface area
KTS	Klippel–Trenaunay syndrome
ASD	Atrial septal defect
PHACES	Posterior fossa brain malformations, hemangiomas, arterial anomalies, cardiac defects, eye anomalies, and sternal clefting/supraumbilical raphe
